# Microbiome Profiling of Biofilms Formed on d-PTFE Membranes Used in Guided Bone Regeneration

**DOI:** 10.3390/microorganisms13112478

**Published:** 2025-10-30

**Authors:** Bojana Mohar Vitezić, Barbara Franović, Ira Renko, Davor Kuiš, Gabrijela Begić, Marko Blašković, Dragana Gabrić, Marina Nikolić, Tamara Šoić Vranić, Diana Veljanovska, Olga Cvijanović Peloza

**Affiliations:** 1 Department of Microbiology and Parasitology, Faculty of Medicine, University of Rijeka, Braće Branchetta 20/1, 51000 Rijeka, Croatia; gabrijela.begic@medri.uniri.hr; 2 Department of Clinical Microbiology, Clinical Hospital Centre Rijeka, Krešimirova 42, 51000 Rijeka, Croatia; 3 Department of Anatomy, Faculty of Medicine, University of Rijeka, Braće Branchetta 20/1, 51000 Rijeka, Croatia; barbara.franovic@uniri.hr (B.F.); marina.nikolic@medri.uniri.hr (M.N.); tamara.soic.vranic@medri.uniri.hr (T.Š.V.); diana.veljanovska@uniri.hr (D.V.); olga.cvijanovic@medri.uniri.hr (O.C.P.); 4 Laboratory for Computational Biology and Translational Medicine, Division of Electronics, Ruđer Bošković Institute, 10000 Zagreb, Croatia; ira.renko@irb.hr; 5 Department of Periodontology, Faculty of Dental Medicine Rijeka, Univeristy of Rijeka, 51000 Rijeka, Croatia; davor.kuis@fdmri.uniri.hr; 6 Clinical Hospital Center Rijeka, 51000 Rijeka, Croatia; 7 Department of Oral Surgery, Faculty of Dental Medicine Rijeka, University of Rijeka, Krešmirova 40/42, 51000 Rijeka, Croatia; marko_blaskovic@yahoo.com; 8 Dental Clinic Dr. Blašković, Linićeva 16, 51000 Rijeka, Croatia; 9 Department of Oral Surgery, School of Dental Medicine, University of Zagreb, Gundulićeva 5, 10000 Zagreb, Croatia; dgabric@sfzg.hr; 10 Department of Dental Medicine, Clinical Hospital Centre Zagreb, 10000 Zagreb, Croatia; 11 Department of Physiotherapy, Faculty of Health Studies, University of Rijeka, Viktora cara Emina 5, 51000 Rijeka, Croatia

**Keywords:** biofilm, microbiome, membrane, metagenomics

## Abstract

In guided bone regeneration (GBR) procedures, d-PTFE membranes are often used as a barrier to promote alveolar ridge regeneration. The aim of this randomized clinical trial was to examine the microbial diversity and structure of biofilms on two types of d-PTFE membranes, Permamem^®^ and Cytoplast™, over four-week oral cavity exposure periods. Bacterial biofilm analysis was performed using 16S rRNA next-generation sequencing (NGS) on 36 samples (20 Permamem^®^ and 16 Cytoplast™). The results showed significant differences in the microbial profiles: Cytoplast™ membranes showed reduced microbial diversity and an enhanced proportion of pathobionts like *Selenomonas*, *Segatella*, *Fusobacterium* and *Parvimonas*, which are associated with periodontal and peri-implant diseases and alveolar bone loss. Permamem^®^ membranes promoted colonization by bacteria associated with healthy oral conditions, such as the genera *Streptococcus*, *Kingella* and *Corynebacterium*. Overall, our results showed that Cytoplast™ membranes generate a specific type of biofilm, leading to reduction in health-related bacterial species and facilitating growth conditions for dysbiosis shift. Further research and patient follow-ups are essential to thoroughly evaluate the clinical implications of different d-PTFE membranes used in guided bone regeneration.

## 1. Introduction

The oral cavity contains a complex and diverse microbial ecosystem comprising over 700 bacterial species that exist in a state of dynamic equilibrium. In oral health, this microbiome is predominantly colonized by aerobic and facultative anaerobic bacteria, such as various species of *Streptococcus* spp., *Actinomyces* spp., *Rothia* spp., *Fusobacterium* spp., *Neisseria* spp., *Haemophilus* spp. and *Leptotrichia* spp. Specific sites are colonized with different proportions of microaerophilic and anaerobic bacteria like *Peptostreptococcus* spp., *Veillonella* spp., *Segatella* spp., *Campylobacter* spp., *Lactobacillus* spp., *Bifidobacterium* spp., *Corynebacterium* spp., *Propionibacterium* spp., *Desulfobacter* spp., *Selenomonas* spp. and *Treponema* spp. [1]. These commensal microorganisms play a key role in maintaining oral homeostasis through competitive restriction of pathogens, modulation of the immune system and formation of protective biofilms. Disruption of oral microbiome homeostasis and development of dysbiosis may cause various diseases and also impair tissue and bone regeneration. Microbial succession proceeds in clearly defined phases from initial colonization by aerobic Gram-positive bacteria, through a transitional phase with anaerobic opportunists, to a late phase dominated by anaerobic pathogens associated with periodontal and peri-implant diseases and impaired healing [2]. The microbial dynamics during this period may significantly affect the outcome of regeneration, including implant integration and volumetric bone restoration.

Guided bone regeneration (GBR) is a standardized method in dental medicine that enables the preservation and restoration of the alveolar ridge, especially in preparation for implant placement. A key component of this method is the use of barrier membranes that physically prevent the migration of fibroblast and epithelial cells into the regeneration area, allowing osteoprogenitor cells to grow without interference [3]. Dense polytetrafluoroethylene (d-PTFE) membranes, such as Cytoplast™ and Permamem^®^, are increasingly used due to their low porosity, resistance to bacterial penetration and suitability for clinical application without primary wound closure [4,5]. These membranes allow predictable regeneration, even in cases where they are exposed to the oral cavity, with minimal risk of infection and graft failure [6].

Despite these advantages, the formation of dental biofilm on the surface of d-PTFE membranes, where they provide specific niche for bacterial colonization, remains a clinical challenge. In clinical settings, premature or excessive colonization of the membrane surface by pathogenic microorganisms may lead to disrupted regeneration, including membrane exposure, inflammation or graft failure [7]. Bacterial adhesion depends on the microstructural characteristics of the membrane, including its degree of crystallinity, nanoscale roughness and topological artifacts. The lower nanoroughness of the Cytoplast™ membrane results from its higher crystallinity (78.6%), which results in stronger bacterial adhesion compared to the lower crystallinity (34.2%) of the Permamem^®^ membrane [8,9]. A recent randomized clinical trial found that membranes with a rougher inner surface, such as Cytoplast™, exhibit higher bacterial adhesion (visualized with electron microscopy) of the microbiome compared to Permamem^®^ membranes [8,9]. Specific niches created around specific membrane types represent major researching and therapeutic challenges.

Peri-implantitis is a biofilm-mediated inflammatory disease that affects the tissues around dental implants—peri-implant mucosa and alveolar bone. If left untreated, it causes gradual bone loss and may lead to implant failure. The peri-implant niche supports a diverse microbial population, with anaerobic Gram-negative bacteria playing an important role. *Porphyromonas gingivalis*, *Tannerella forsythia*, *Treponema denticola* and *Fusobacterium nucleatum* are important periopathogens that release virulence factors that cause tissue damage and regulate host immunological responses [10]. Furthermore, Gram-positive organisms such as *Staphylococcus epidermidis* and enteric bacteria have been found in higher abundance in peri-implantitis locations than in healthy implants, indicating a diverse microbial dysbiosis driving disease progression. The analytical advantages of next-generation sequencing highlight the crucial role which biofilm architecture and interspecies interactions have in the pathophysiology of peri-implantitis, by enabling thorough profiling of microbial changes from peri-implant health to peri-implant diseases. Comprehending these microbiological boundaries is crucial for precise diagnosis as well as the creation of focused treatment methods, such as host-modulation techniques, photodynamic therapy and mechanical debridement with adjunct antimicrobials [11].

The formation and colonization of biofilms on different surfaces like dental implants, d-PTFE membranes and others involves a different process compared to natural teeth. When saliva comes into contact with the implant surface, salivary proteins immediately adhere and form an acquired pellicle. This pellicle differs from the enamel pellicle containing α-amylase, mucins, proline-rich proteins and secretory IgA, while lacking low-molecular weight mucins and cystatins. Bacterial colonization begins in areas protected from shear forces, driven by electrostatic, Van der Waals and hydrophobic interactions that place bacteria close to the pellicle-coated surface. Initial adhesion becomes irreversible once bacteria bind to the pellicle proteins. Early biofilm development is dominated by facultative Gram-positive cocci, rods and bacilli, with limited Gram-negative anaerobes. *Streptococcus sanguinis* and *Actinomyces naeslundii* are the primary colonizers, and *Streptococcus* spp. rapidly establish themselves on exposed implant surfaces. Titanium surfaces create a unique microenvironment that alters ecological succession—early peri-implant biofilms show reduced levels of *A. naeslundii* and marked co-aggregation with *Veillonella* spp. As early colonizers multiply, they change the environment and facilitate the adhesion of secondary colonizers through co-aggregation. After days of maturation, a microbial change occurs: the number of Gram-negative cocci, spirochetes and motile bacilli increases, especially in the submucosal areas. This change—combined with immune dysfunction—can cause peri-implant mucositis, a precursor to peri-implantitis [12].

Gram-positive cocci and non-motile bacilli predominate in the healthy peri-implant microbiome, while anaerobic Gram-negative organisms are detected in minor portions. Commensal microorganisms like *Segatella multiformis*, *Kingella oralis*, *Actinomyces massiliensis* and *Lautropia mirabilis* help maintain community homeostasis by restricting adhesion of possible pathogens in the supramucosal niche. In addition to *Actinomyces naeslundii*, *A. oris*, *A. meyeri* and *A. massiliensis*, the submucosal sulcus is home to a more complex consortium that includes *Streptococcus sanguinis*, *S. salivarius* and *S. oralis, Veillonella dispar*, *Rothia aeria*, *Rothia dentocariosa*, *Capnocytophaga sputigena*, *Haemophilus parainfluenzae*, *Corynebacterium matruchotii*, *Leptotrichia* sp., *Neisseria* sp., *Mycoplasma salivarium*, *Eikenella corrodens* and non-pathogenic *Fusobacterium* sp. Low concentrations of the traditional periodontal pathogens *Aggregatibacter actinomycetemcomitans*, *Porphyromonas gingivalis*, *Fusobacterium nucleatum*, *Tannerella forsythia*, *Treponema denticola*, *Segatella intermedia*, *Parvimonas micra* and *Streptococcus intermedius* remain in healthy peri-implant sulci without endangering implant stability in well-maintained individuals, even in the absence of clinical inflammation [12]. Members of the *Actinomycetia class*, bacilli like *Granulicatella*, *Gammaproteobacteria* (like *Vibrio)*, *Epsilonproteobacteria* (like *Campylobacter*) and genera found only in health, like *Filifactor*, *Bradyrhizobium*, *Dialister*, *Paludibacter*, *Staphylococcus*, *Acinetobacter* and *Propionibacterium* are among the health-associated taxa enriched at these sites. A core network composed of *Firmicutes*, *Spirochaetes* and *Bacteroidetes* underlies both supramucosal and submucosal communities. The preservation of peri-implant health is determined by the balance of site-specific microbial interactions [13].

### 1.1. Peri-Implant Mucositis Microbiome

Increased cocci, motile bacilli and spirochetes are indicators of peri-implant mucositis, an intermediate microbiological condition between peri-implantitis and health. Mucositis sites contain elevated levels of periodontal pathogens, including *Segatella intermedia*, *P. gingivalis*, *T. forsythia*, *T. denticola* and *Segatella denticola*. Recent taxonomic research (2023) has subdivided the genus *Prevotella* into seven genera, introducing four new genera named *Segatella*, *Hoylesella*, *Leyella* and *Palleniella* [14]. After implant placement, patients with a history of periodontitis exhibit submucosal biofilms that mirror their initial periodontal flora and are enriched in enteric rods like *Fusobacterium* spp., *P. gingivalis*, *T. forsythia*, *Campylobacter rectus*, *Dialister pneumosintes* and *Peptostreptococcus micros*, according to case studies. According to recent research, the genera *Segatella and Fusobacterium* may be biomarkers of mucositis, indicating a tendency in people who have previously had periodontal disease [13].

### 1.2. Peri-Implantitis Microbiome

Deepened pockets and anaerobic environments that support intricate bacterial, fungal and viral biofilms lead to peri-implantitis. Gram-negative anaerobes as *A. actinomycetemcomitans*, *Capnocytophaga* spp., *F. nucleatum*, *S. intermedia*, *T. forsythia*, *T. denticola*, *T. maltophilum* and *P. gingivalis* dominated the submucosal flora, which was initially compared to periodontitis. More opportunists, such as *Staphylococcus aureus*, *S. epidermidis*, *Pseudomonas aeruginosa*, *Peptostreptococcus species*, *Neisseria species*, *Porphyromonas endodontalis*, *Lactococcus lactis*, *Filifactor alocis*, *Escherichia coli*, *Enterobacter species*, *Helicobacter pylori* and *Candida* sp., are discovered by sophisticated molecular techniques. One-third of locations have Epstein–Barr virus and human cytomegalovirus co-infections, which may reduce local immunity and promote pathogenic development [14].

Considering mentioned different microbiome biofilm architectures, the concept of personalized regenerative therapy based on the patient’s microbiological profile is gaining interest. Microbiome analysis prior to or during therapy may potentially guide the selection of the optimal membrane material or the implementation of additional antimicrobial strategies [15,16].

Elucidating these microbial dynamics may also contribute to the development of evidence-based protocols for membrane selection and timing of surgical interventions, ultimately improving patient outcomes in regenerative dentistry.

The aim of this study was to analyze microbial diversity and structural composition of mature biofilm on two different d-PTFE membranes during a four-week period following guided bone regeneration. Bacterial biofilm was analyzed using 16s rRNA next-generation sequencing (NGS) in order to identify microbial dynamics and its dysbiosis potential.

## 2. Materials and Methods

### 2.1. Study Design and Patient Selection

This study included patients who required at least one tooth extraction and subsequent implant placement. Of the 56 patients initially evaluated, 39 met the inclusion criteria and were randomly assigned to one of two study groups. Detailed protocol, inclusion/exclusion criteria, sample size and other study factors were described in the article by Franović et al. [8]. Individuals were assigned to intervention groups based on the web-based randomization process. By the end of our study, we managed to collect and analyze 36 d-PTFE, 20 Permamem^®^ and 16 Cytoplast™ membranes from patients after a four-week healing period, following guided bone regeneration. In our study, 39 membranes, from 39 patients, were extracted. Membranes extracted from 3 excluded patients had insufficient (below the detection limit) DNA isolate concentrations and were consequently eliminated from NGS analysis. These exclusions were distributed across the two membrane types and were not disproportionately associated with either Permamem^®^ or Cytoplast™ membranes, minimizing the risk of systematic bias associated with membrane type.

The study comprised men and women over the age of 18 who were in good general health and had tooth extraction grounds due to vertical or horizontal fractures extending 2 mm or more apically from the gingival border, failed endodontic or other conventional therapies. Exclusion criteria were as follows: patients with head and neck irradiation, those undergoing intravenous or oral bisphosphonate therapy, patients with untreated periodontal disease or uncontrolled diabetes, patients on immunosuppressive or long-term corticosteroid therapy, pregnant and lactating women and patients who smoked more than 10 cigarettes per day.

### 2.2. Grafting Surgery

Before surgery, all subjects had their plaque and calculus removed mechanically and were instructed on proper oral hygiene. An oral antibiotic (Klavocin^®^ bid 1000 mg, Pliva, Zagreb, Croatia) was given an hour before surgery. Additionally, patients rinsed with a 0.2% chlorhexidine digluconate solution (Parodontax^®^ 0.2%, Brentford, London, UK) prior to surgery.

A socket grafting operation was conducted immediately after tooth extraction in both groups using a composite bone graft including 50% autogenous bone and 50% bovine xenograft (Cerabone^®^, Botiss Biomaterials GmbH, Zossen, Germany). Following graft placement, the grafted site was coated with one of two membranes based on their pre-assigned group as follows:

Group P: The grafted site was coated with a d-PTFE membrane (Permamem^®^, Botiss Biomaterials GmbH, Zossen, Germany).

Group C: The grafted site was coated with an alternate d-PTFE membrane (Cytoplast™, Osteogenics Biomedical, Lubbock, TX, USA).

Four weeks after extraction and grafting, the d-PTFE membrane was removed in both groups, leaving the pseudoperiosteum covering the crestal section of the extraction socket exposed to promote healing. After six months of healing, implants were placed in the prepared spot. Detailed protocol was described in the article by Franović et al. [8].

### 2.3. Ethical Considerations

This RCT followed the guidelines outlined in the Declaration of Helsinki. The Ethics Committee of the Faculty of Medicine at the University of Rijeka (class: 003-05/20-1/151, number. 2170/29-02/1-20-2) accepted the study of human volunteers who gave informed consent. This study followed ethical guidelines and was accepted as an RCT, which is registered on ClinicalTrials.gov (NCT06694844). This study followed the CONSORT standards for reporting randomized controlled trials. All participants provided informed permission and voluntarily agreed to participate after being fully briefed on the study’s methods and requirements.

### 2.4. Sonication and DNA Extraction

Following membrane extraction, the d-PTFE membranes were placed in Transystem Amies w/o Ch (Copan) and promptly transferred to the Faculty of Medicine Rijeka for further examination. Upon arrival, the membranes were rinsed 3× and placed into 2.5 mL saline and transferred to an ultrasonic bath (BANDELIN electronic GmbH & Co. KG, Berlin, Germany) under standardized conditions of 3 min/40 kHz to release d-PTFE membrane biofilm into the sonicate. Approximately 1 mL of the resulting sonicate was collected and frozen at −80 °C (Heraeus Group, Hanau, Germany) for subsequent DNA extraction and next-generation sequencing. Extractions of DNA were performed on an automated nucleic acid extraction system SACACE—SaMag-12 (Sacace Biotechnologies, Como, Italy) with SaMag Bacterial DNA Extraction kit -SM006 (Sacace Biotechnologies, Como, Italy). Prior to DNA extraction, sonicates were centrifugated for 10 min at 15,000 rpm and the supernatant was removed with 200 µL of sediment left at the bottom of the tube. Afterward, 200 µL of lysis buffer (20 mM Tris/HCL, 2 mM EDTA, 1% Triton X-100, pH 8) was added to sediment with 50 µL of freshly prepared lysozime (0.02 g lysozime in 500 µL 20 mM TrisHCl). The mixture was incubated in a thermomixer for 45 min at 37 °C and 900 rpm. After 45 min of incubation, 50 µL of proteinase K was added and further incubated for 30 min at 56 °C and 900 rpm. Following described pretreatment of sonicates, the entire volume of liquid was transferred into sample tubes at SaMag-12 instrument (Sacace Biotechnologies, Como, Italy) and extraction was performed automatically according to SaMag Bacterial DNA Extraction kit protocol with settings for sample volume 400 µL and elution volume 50 µL. After DNA extraction, concentrations of DNA were determined with Qubit fluorometer (TermoFisher Scientific, Waltham, MA, USA) with the Qubit DNAHS kit (TermoFisher Scientific, Waltham, MA, USA) according to the manufacturer’s protocol.

### 2.5. 16S rRNA Amplification and Next-Generation Sequencing Analysis

Microbial diversity and abundance in supragingival dental biofilms of the examined groups were assessed by sequencing of bacterial 16s rRNA gene using next-generation sequencing (NGS) on the Illumina platform. Thirty-six DNA isolate samples from membrane sonicates (20 membrane samples from the Permamem^®^ group and 16 membrane samples from the Cytoplast™ group) were sent to the Molecular Research Laboratory (MRDNA) in Texas, USA, where amplicon sequencing was performed using a set of 341F (5′-CCTAYGGGRBGCASCAG-3′) and 806R (5′-GGACTACNNGGGTATCTAAT-3′) primers. The resulting sequencing data were obtained from “Illumina’s BaseSpace Sequence Hub” as paired-end, demultiplexed fastq files. Data processing was carried out using the software package Quantitative Insights Into Microbial Ecology 2 (QIIME2) (2024.2) [17]. The workflow included filtering and denoising using the DADA2 (2024.2) program (integrated within QIIME2), concatenation of the sequences and control for the presence of possible chimeras [18]. For the taxonomic determination of microorganisms, the Naive Bayes classifier tool in QIIME2 was applied, adapted to work with the SILVA database, version 138 [19]. The diversity and richness of all samples were estimated based on beta diversity (Bray–Curtis dissimilarity) using the seaborn, matplotlib, pandas, and matplotlib libraries and Python programming language, version 3.12, within the Pycharm environment (3.12) [20]. Graphical representations were created using RStatistical Software (v4.4.2; R Core Team 2021) using ggplot2 package (3.5.2) and MicrobiomeAnalyst 2.0 web-based platform [21].

### 2.6. Statistical Analysis

Data collected during the study were analyzed using conventional descriptive statistical methods. The normality of data distribution was assessed using QQ plots. Most analyses were performed using the Python programming language (version 3.12), within the PyCharm environment, utilizing the pandas, seaborn, and matplotlib libraries [20,22]. Additional statistical analyses were conducted using the R programming language (version 4.4.2), specifically the ALDEx2, bioconductor package (analysis of differential abundance taking sample and scale variation into account (1.38) [23]. This method relies on Monte Carlo sampling from the Dirichlet distribution and enables robust estimation of differential taxon abundance in next-generation sequencing (NGS) data, with automatic application of Benjamini–Hochberg (BH) correction. Results were considered statistically significant when the we.eBH-Wilcoxon *p*-value (Benjamini–Hochberg corrected) was below 0.05.

## 3. Results

To assess the diversity and differences in the biofilm microbiome profile between d-PTFE Cytoplast™ and Permamem^®^ membrane-sonicated DNA samples, NGS results were comparatively analyzed.

Permamem^®^ membrane group included 20 samples marked as M2-M9, M11-M15, M17, M23-M27 and M43.

Cytoplast™ membrane group included 16 samples marked as M16, M28-M35, M37-M39, M42 and M46-M48.

To determine whether there were significant differences between the groups, the most abundant (presented in the core microbiome) and rarest group-specific and disease-specific bacterial genera and species were analyzed. After processing, the total number of reads across all samples was 1,099,274 with an average number of reads for Cytoplast™ samples of 377,862, and for Permamem^®^ samples of 351,592 reads. Taxonomic classification of the sequences identified 284 species linked to a total of 13 phyla across samples. Among them, 275 were related to Permamem^®^ samples and 242 to Cytoplast™ samples.

**Figure 1:**
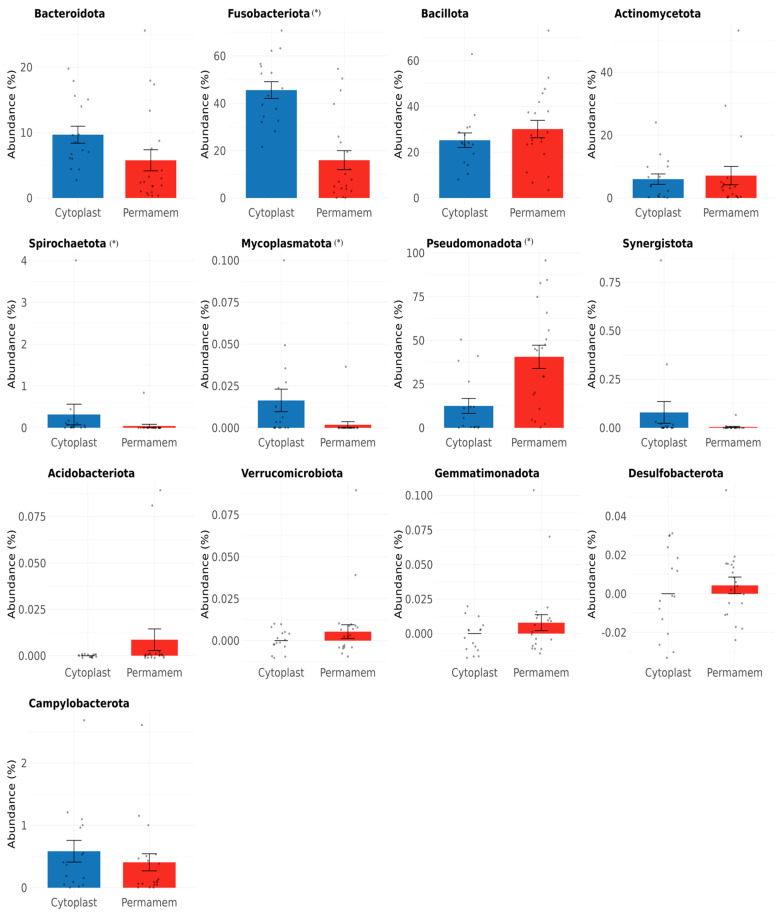
Bacterial species detected at the phylum level in the Cytoplast™ and Permamem^®^ group. * indicates *p* < 0.05.

At the phylum level, clear differences were observed between the two groups (Figure 1).

Of the 13 bacterial phyla detected in both groups, the most dominant in the Permamem^®^ group were *Pseudomonadota* (40%), *Bacillota* (30%) and *Fusobacteriota* (16%), while in Cytoplast™ group, *Fusobacteriota* was most dominant (46%), followed by *Bacillota* (25%) and *Pseudomonadota* (13%). In the Permamem^®^ group, *Actinomycetota* (7%) and *Bacteroidota* (6%) were the less abundant phyla, whereas in the Cytoplast™ group, *Bacteroidota* (10%) and *Actinomycetota* (6%) showed lower abundance. Other phyla, including *Spirochaetota*, *Mycoplasmatota*, *Synergistota*, *Acidobacteriota*, *Verrucomicrobiota*, *Gemmatimonadota*, *Desulfobacterota* and *Campylobacterota*, were observed only at very low relative abundances (<1%) and showed inconsistent presence across groups.

**Figure 2:**
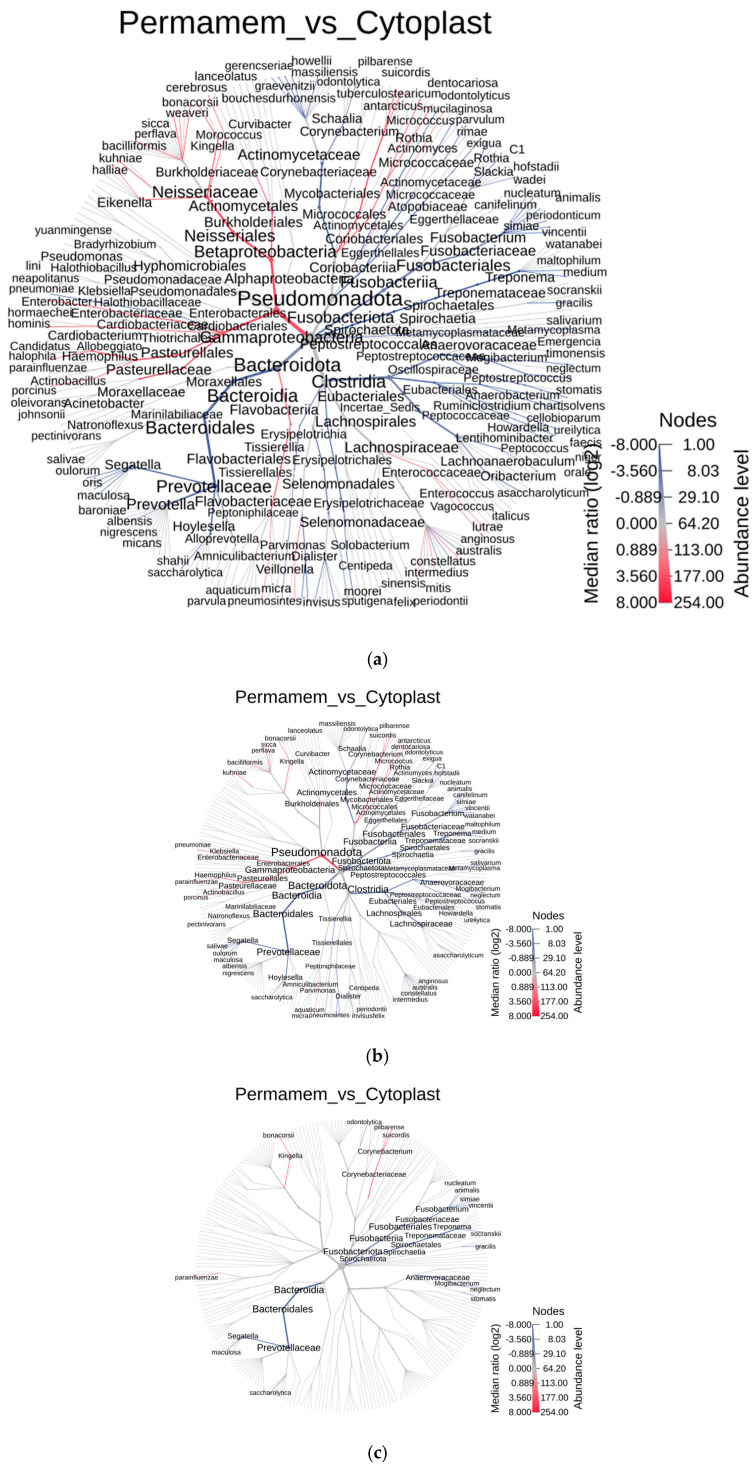
Heat tree at the level of species, (**a**) *p* < 0.05, (**b**) *p* < 0.01, (**c**) *p* < 0.001.

To further explore the differences between the two groups at lower taxonomic levels, a heat tree analysis was performed using the MicrobiomeAnalyst 2.0 web tool [21] (Figure 2). Differences between groups were defined using the Wilcoxon test at three significance thresholds (*p* < 0.05, *p* < 0.01, *p* < 0.001) to strengthen the validity of the findings. In the resulting heat trees, nodes represent species, node size represents abundance, and node color indicates the significance of the difference between groups. **Blue** stands for enhanced species presence in **Cytoplast™** and **red** stands for enhanced species presence in **Permamem^®^** group).

As shown in Figure 2 and Table 1, the number of differing species at a significance threshold of *p* < 0.05 was 28. Differences remain evident, even at the more stringent *p* < 0.01 value, with 23 species and at the most stringent cut off *p* < 0.001, the two groups differ in 15 species.

Of 23 species, which differed between groups, detected at *p* < 0.01 value, the majority in the Cytoplast™ group belonged to the genera *Fusobacterium* and *Segatella*, whereas in the Permamem^®^ group, the dominant species belonged to genera *Kingella*, *Klebsiella* and *Corynebacterium* (Figure 2b,c and Table 2). At the most stringent cut off of *p* < 0.001, the two groups differ in 15 species belonging to the genera *Segatella*, *Haemophilus*, *Kingella*, *Fusobacterium*, *Corynebacterium*, *Treponema* and *Mogibacterium*, with all listed taxa except *Kingella* and *Corynebacterium* being more abundant in the Cytoplast™ group compared to Permamem^®^.

To assess the potential presence of false positive results, ALDEx2 analysis (analysis of differential abundance taking into account sampling variation) was performed. During the analysis, the Benjamini–Hochberg correction was applied to control for false positive results. Genera and species with a *p*-value (Wilcoxon Effect—Benjamini–Hochberg) of less than 0.05 were considered statistically significant. The test results are presented in Table 2a,b.

Based on the results, among the statistically significant bacterial genera, the genus *Kingella* shows the highest positive difference in abundance between the Permamem^®^ and Cytoplast™ groups, with its presence being significantly more pronounced in the Permamem^®^ group. In contrast, the genera *Segatella* and *Fusobacterium* showed a statistically significant increase in the Cytoplast™ group, indicating distinct microbial profiles between the analyzed groups (Table 2a).

A similar trend was observed at the species level, where *Kingella bonacorsii* was identified as the species with the highest positive difference in abundance in favor of the Permamem^®^ group. On the other hand, species of the genus *Fusobacterium* exhibit a higher relative abundance in the Cytoplast™ group compared to Permamem^®^, further supporting the presence of a distinct microbial dominance within that group (Table 2b).

Beta diversity analysis revealed no significant differences between the two groups (*p* = 0.5).

**Figure 3:**
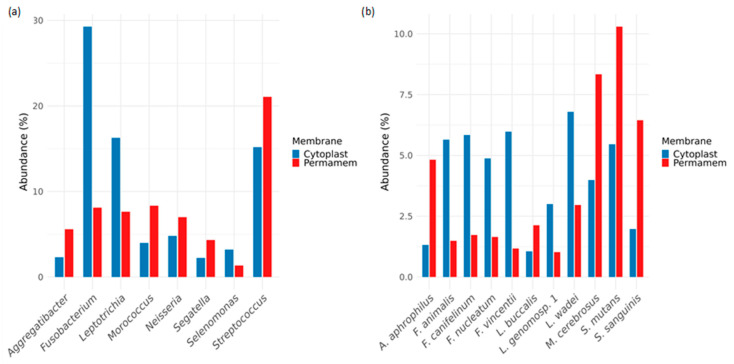
Core microbiome bacteria on the level of (**a**) genus and (**b**) species.

**Figure 4:**
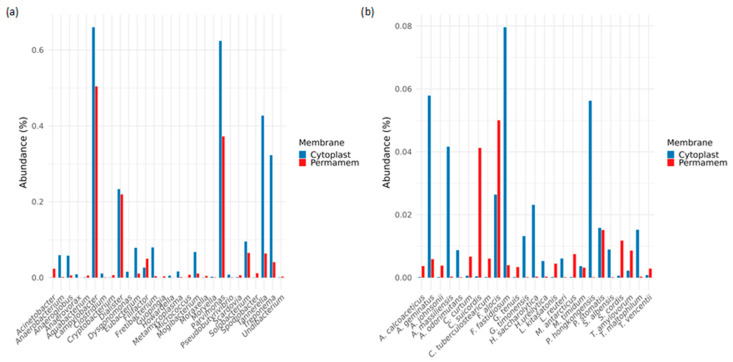
Rare and uncommon bacteria on the level of (**a**) genus and (**b**) species.

In order to further explore detected bacteria, the most abundant bacteria of the core microbiome were identified at the genus and species level (Figure 3), as well as for the rare bacteria (Figure 4).

The relative abundances of bacterial taxa at the genus level differed substantially between the Cytoplast™ and Permamem^®^ groups. The Cytoplast™ group was particularly enriched in the genera *Fusobacterium* (~30%), *Leptotrichia* (~17%) and *Streptococcus* (~15%). In contrast, the Permamem^®^ group exhibited a distinct distribution, with *Fusobacterium* (~8%) and *Leptotrichia* (~7%) being less represented, while *Streptococcus* (~21%) emerged as the dominant genus (Figure 3a).

At the species level, the profiles were similarly non-uniform. In the Permamem^®^ group, the most abundant species included *Streptococcus mutans* (~10.5%), *Morococcus cerebrosus* (~8.5%) and *Streptococcus sanguinis* (~6.5%), followed by *Aggregatibacter aphrophilus* (~4.8%) and *Leptotrichia wadei* (~3%). The same species were present in the Cytoplast™ group, but at markedly different relative abundances: *S. mutans* (~5.5%), *M. cerebrosus* (~4%), *S. sanguinis* and *A. Aphrophilus,* with *L. wadei* detected at low abundance (Figure 3b).

Consistent with Figure 3a, the Cytoplast™ group was dominated by the *Fusobacterium* species (*F. Nucleatum*, *F. animalis*, *F. canifelinum*, *F. vincentii* and *F. nucleatum*), which in Permamem^®^ samples were detected only at <2.5% relative abundance (Figure 3b).

With respect to the less abundant but noteworthy bacterial genera, several taxa were relatively enriched in the Cytoplast™ group, including *Campylobacter* (0.65%), *Dialister* (0.22%), *Treponema* (0.31%), *Parvimonas* (0.63%) and *Tannerella* (0.42%). Among these genera, *Campylobacter* (0.5%) was the most abundant in the Permamem^®^ group, followed by *Parvimonas* (0.38%) and *Dialister* (0.21%). *Treponema* and *Tannerella*, as well as the majority of genera depicted in Figure 4a, were detected at levels below 0.1% (Figure 4a).

At the species level (Figure 4b), the Permamem^®^ group was characterized by the predominance of *Corynebacterium suiscordis* (0.041%) and *Filifactor alocis* (0.05%), which were only marginally detected in the Cytoplast™ group (0.001% and 0.008%, respectively). In contrast, the Cytoplast™ group was dominated by several rarely represented species, including *Anaeroglobus geminatus* (0.059%), *Actinomyces massiliensis* (0.042%), *Filifactor fastidiosum* (0.08%) and *Pseudostreptobacillus honkongensis* (0.058%).

**Figure 5:**
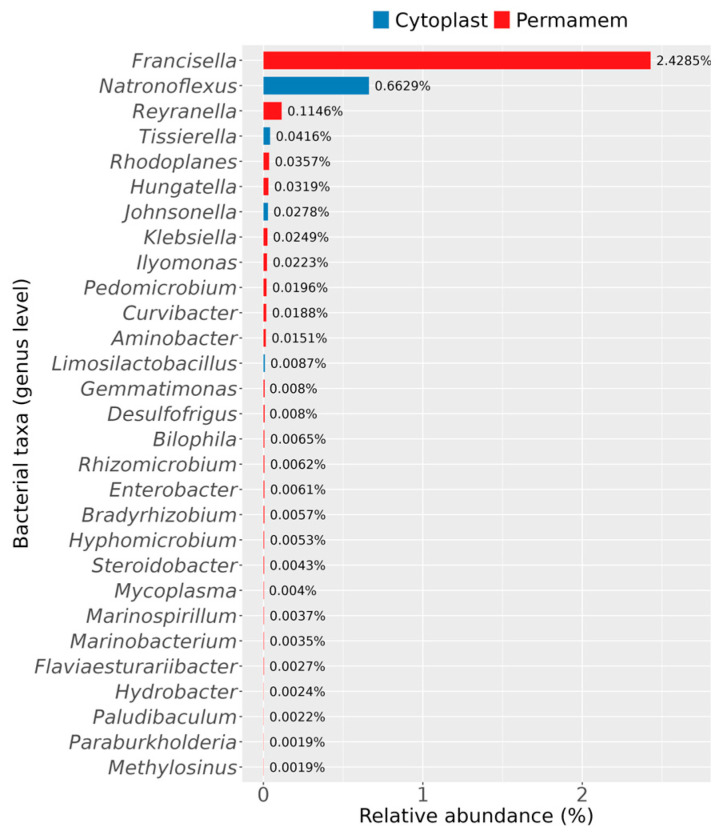
Bacterial genera specific for either Permamem^®^ or Cytoplast™ samples.

Furthermore, it was observed that some bacterial genera were uniquely detected in only one of the two groups, as illustrated in Figure 5.

With respect to bacteria specific to each group, the Permamem^®^ samples contained a greater number of uniquely identified genera compared to the Cytoplast™ group (Figure 5). The most striking differences were observed for the genera *Francisella*, *Reyranella* and *Natronoflexus*. *Francisella* dominated in the Permamem^®^ samples, with a substantial relative abundance of 2.4%, while *Reyranella* (0.1%), *Rhodoplanes* and *Hungatella* (0.04%) were detected at lower levels. In contrast, the Cytoplast™ samples contained members of the genera *Natronoflexus* (0.7%), *Tissierella* (0.04%), *Johnsonella* (0.2%) and *Limosilactobacillus*, none of which were detected in the Permamem^®^ group. Other genera, not explicitly mentioned here but displayed in Figure 5, belonged to the Permamem^®^ group and were present at very low abundances (<0.1%).

Furthermore, among the detected taxa, particular attention was given to species of relevance in the context of periodontitis, implant and bone loss. Nine bacteria genera that were observed included *Dialister*, *Filifactor*, *Fretibacterium*, *Mogibacterium*, *Parvimonas*, *Peptostreptococcus*, *Selenomonas*, *Shuttleworthia* and *Solobacterium*.

**Figure 6:**
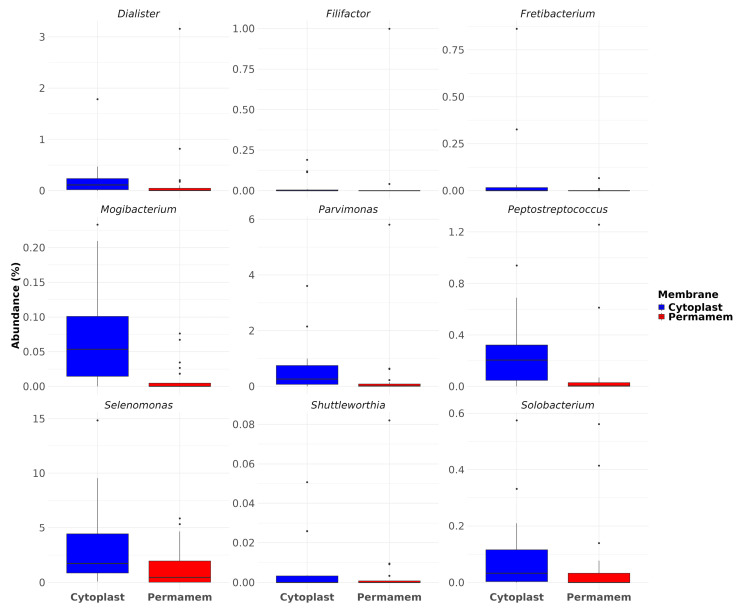
Dysbiosis and disease-related bacterial genera.

Analysis of their relative abundances revealed that Cytoplast™ samples exhibited consistently higher levels of all selected genera compared to Permamem^®^, most prominently *Selenomonas*, exhibiting the strongest enrichment, reaching values above 10% in some samples, while being significantly reduced in Permamem^®^ (2%). Similarly, *Parvimonas* and *Peptostreptococcus* displayed consistently higher levels in Cytoplast™samples, with Permamem^®^ showing only minimal representation(Figure 6). *Mogibacterium* also showed a higher representation in Cytoplast™ (median ~0.05%) but was almost absent in Permamem^®^ samples. Other genera, including *Dialister*, *Fretibacterium*, *Shuttleworthia* and *Solobacterium* followed a similar trend. This observation was further supported by statistical testing: differences in relative abundances were evaluated using the Wilcoxon rank-sum test with Benjamini–Hochberg (BH) correction for multiple comparisons, which confirmed statistically significant differences for *Dialister*, *Mogibacterium*, *Parvimonas* and *Peptostreptococcus*.

## 4. Discussion

The aim of this study was to report a complex microbial composition of mature biofilm on two different d-PTFE membranes exposed to the oral cavity environment for four weeks, following guided bone regeneration in vivo. The composition of bacterial biofilm is niche-specific and location-dependent. 16s rRNA sequencing and novel bioinformatic pipelines enable detailed analysis and insights in composition of microbial colonizers on d-PTFE membranes, which present a specific niche for development of unique microbial communities. All patients in our study received prophylactic antibiotics prior to surgery according to standard clinical protocols to reduce the risk of postoperative infections. We acknowledge that systemic exposure to antibiotics can alter the composition of the oral microbiome and potentially influence subsequent biofilm formation. However, all participants received the same antibiotic regimen under controlled conditions; any effects on the oral microbiome are expected to be relatively uniform across study groups and minimize variability between groups.

The different membrane-type surfaces were, as expected, colonized by a perplexing palette of diverse microorganisms. Bacterial adhesion depends on the different microstructural characteristics of each used Cytoplast™ (C) or Permamem^®^ (P) membrane (degree of crystallinity, nanoscale roughness or topological artifacts). Comparison of overall 16s rRNA sequence read number was conducted and higher values were detected on C membranes, suggesting a higher rate of adhesion and bacterial accumulation compared to P membranes. Results of scanning electron microscopy (SEM) presented in a previously published article (8), showed denser extracellular matrix and bacterial clusters on C membranes compared to P membranes, supporting the finding of higher bacterial adhesion and accumulation on C membranes.

When comparing species diversity, 275 species were detected on P membranes compared to 242 species detected on C membranes; reduced diversity of species in oral microbiota was already detected and described as dysbiosis which represent the shift in balance of a specific niche microbial community [24].

Previously demonstrated significant structural and surface differences between dense PTFE Cytoplast™ and Permamem^®^ membranes [9] suggest that membrane material and topography may influence early microbial selection and could have contributed to the dysbiotic pattern observed in Cytoplast™ biofilms. Dysbiosis may diminish species contributing to the healthy balance in specific biofilms and contribute to the overgrowth of pathobionts and oral pathogens, leading to inflammation, periodontitis, tooth, implant and bone loss.

Beta diversity measures overall community composition differences between groups and our results revealed no significant difference between P and C membrane types. Differential abundance analysis such as ALDEx2 evaluates each taxon individually, identifying specific taxa whose relative abundances differ between groups, even if these changes do not substantially alter the overall community structure like described with beta diversity.

Considering differences between researched membrane types, dominance of *Pseudomonadota* (40%), *Bacillota* (30%) and *Fusobacteriota* (16%) was detected in the P group while in the C group, *Fusobacteriota* was in majority (46%), followed by *Bacillota* (25%) and *Pseudomonadota* (13%). These findings may indicate differing biofilm maturation states between membrane types. *Fusobacteriota* is consistent with more mature anaerobic biofilms, whereas *Pseudomonadota* may reflect earlier colonization stages, suggesting slower and delayed biofilm formation on P membranes. Since a single time point was analyzed, this interpretation remains hypothetical. Longitudinal sampling would provide valuable insights into biofilm succession; unfortunately, in our study, we could only have a single endpoint of four weeks, which corresponds to the required clinical timeframe for removal of the d-PTFE membrane after guided bone regeneration. This time point provides insight primarily into the state of the mature biofilm and the composition of its microbial community. Earlier removal of membranes would interrupt the bone healing process and disrupt patient recovery, so it was impossible to perform it in a clinical trial.

The P and C groups also differed among prevalent genera: In the P group, *Streptococcus* (21%), *Haemophilus* (10%), *Fusobacterium* (8%) and *Neisseria* (7%) were detected. On the other hand, the C group exhibited a high abundance of *Fusobacterium* (29%) and *Streptococcus* (15%) but differed in the remaining dominant taxa. Considering detected species, the P group was dominated by *Streptococcus mutans* (10%), *Haemophilus parainfluenzae* (10%) and *Moraxella cerebrosus* (8%) while in the C group, species of the *Fusobacterium* genus prevailed: *F. animalis* (6%), *F. canifelinum* (6%) and *F. vincentii* (6%). These results suggest slower biofilm formation on P membranes with higher abundance of *Streptococcus* spp. while a higher *Fusobacterium* spp. level indicates developed maturity of biofilm and shift to thicker anaerobic conditions on the C membrane, leading to possible dysbiosis [24]. Fusobacterium acts as a “bridge” organism facilitating co-aggregation with late colonizers and accelerates the maturation of pathogenic biofilm [25,26,27,28,29,30,31].

Statistically significant bacterial genera with enhanced presence in the P membrane group were as follows: *Kingella*, *Corynebacterium*, *Haemophilus* and species: *Kingella bonacorsii* and *Hemophilus parainfluenzae* indicate an enhanced presence of health-associated genera and species which are crucial for pathogen and pathobiont limitation. In contrast, pathogens including genera *Segatella* and *Fusobacterium* with different species presented significantly increased colonization on C membranes compared to P membranes, indicating distinct microbial profiles between the analyzed groups. When analyzing achieved data altogether, one should conclude that the C membrane may generate a microenvironment inside a specific niche, which creates favorable conditions for microbial community shift in dysbiosis direction. Creating such a kind of biofilm on C membranes leads to reduction in health-related bacterial species, creates space and facilitates growth conditions for pathobiont and pathogen species dominance. When analyzing rare bacteria detected on both membrane types, *Francisella* dominated in the P membrane followed by *Reyranella*, *Rhodoplanes* and *Hungatella*. On the C membranes, *Natronoflexus*, *Tissierella* and *Johnsonella* were detected. These bacterial genera detected in different proportions on both membrane types are members of distinct and metabolically different bacteria providing insight into complex and dependent correlations inside specific microbial niche like biofilms created on d-PTFE membranes. Interestingly, the genus *Natronoflexus* was detected mainly on C membranes, suggesting a difference in the biofilm pH with likely more alkaline values at certain time point of biofilm formation. Genera *Selenomonas*, *Parvimonas*, *Peptostreptococcus*, *Dialister*, *Fretibacterium*, *Shuttleworthia* and *Solobacterium* displayed consistently higher levels in the Cytoplast™ samples, confirming higher bacterial genera abundance of relevance in the context of periodontal and peri-implant diseases and alveolar bone loss. Increased abundance of *Peptostreptococcus*, *Selenomonas* and *Parvimonas* in Cytoplast™ biofilms further implicates that C membranes has an enhanced potential for a dysbiotic shift in microbial communities, which may pose a clinical risk for tissue and bone health [28,29,30].

This differential colonization pattern points to a potential role of membrane-specific properties and specific microenvironment in shaping microbial community structure. Early colonization by dysbiotic taxa could affect wound stability, inflammatory processes and bone remodeling. Short-term dysbiosis may have long-term clinical implications despite membrane removal. The clinical implications of various d-PTFE membranes employed in guided bone regeneration merit further comprehensive research. Given that these membranes are typically removed after a four-week period, additional studies are essential to thoroughly assess bone regeneration outcomes and to monitor potential complications, including tissue infections and bone loss.

## 5. Conclusions

Microbial communities adherent on different membranes represent fluid and changing biofilm microenvironments consistent with a wide palette of microorganisms.

When compared, two distinct d-PTFE membranes differed in their temporal biofilm microbiome profiles after a four-week exposure and healing period during guided bone regeneration; the Cytoplast™ membrane represented a better surface for adhesion and bacterial accumulation compared to the Permamem^®^ membrane, considering the higher overall 16s rRNA sequence read number. When analyzing the microbial profile of biofilms adherent on Cytoplast™ or Permamem^®^ d-PTFE membranes, Permamem^®^ membranes generate a biofilm enriched in health-associated bacterial genera and species like *Strepococcus*, *Corynebacterium* and *Kingella*. Cytoplast™ membranes showed reduced microbial diversity and enhanced quantity of pathobionts like Selenomonas and periodontal, peri-implant diseases and alveolar bone loss related bacterial genera, like *Segatella*, *Fusobacterium* and *Parvimonas*. Other disease-related bacterial genera like *Peptostreptococcus*, *Mogibacterium*, *Dialister*, *Fretibacterium*, *Shuttleworthia* and *Solobacterium* presented a similar trend of increased presence in Cytoplast™ membranes. When considering bacterial species which may lead into dysbiosis, *Segatella maculosa* and different *Fusobacterium* species were also more abundant in the Cytoplast™ group. Overall, our result showed that Cytoplast™ membranes generate a specific kind of biofilm, leading to reduction in health-related bacterial species and facilitating growth conditions for dysbiosis shift, pathobiont and pathogen species dominance. Further research and patient follow-ups are essential to thoroughly evaluate the clinical implications of different d-PTFE membranes used in guided bone regeneration.

## Figures and Tables

**Table 1:** Differing bacterial species among Permamem^®^ and Cytoplast™ groups detected at a significance threshold of *p* < 0.05 –0.01, *p* < 0.01–0.001 and *p* < 0.001. **Blue** stands for enhanced species presence in **Cytoplast™** and **red** stands for greater species presence in **Permamem^®^** group).

*p* (<0.05–0.01)		*p* (0.01–0.001)		*p* < 0.001	
Bacterial Species	*p*	Bacterial Species	*p*	Bacterial Species	*p*
*Acinetobacter johnsonii*	0.035	*Treponema maltophilum*	0.005	*Treponema socranskii*	0.00011
*Acinetobacter oleivorans*	0.036	*Streptococus anginosus*	0.001	*Kingella bonacorsii*	1 × 10^−5^
*Actinomyces bouchesdurhonensis*	0.020	*Streptococcus constellatus*	0.006	*Hoylesella saccharolytica*	0.00004
*Actinomyces graevenitzii*	0.034	*Streptococcus porcinus*	0.007	*Haemophilus parainfluenzae*	0.0003
*Actinomyces howellii*	0.011	*Slackia exigua*	0.001	*Fusobacterium vincentii*	0.0002
*Actinomyces gerencseriae*	0.042	*Segatella salivae*	0.004	*Fusobacterium simiae*	0.0002
*Anaerobacterium chartisolvens*	0.015	*Segatella oulorum*	0.002	*Fusobacterium animalis*	0.0003
*Bradyrhizobium yuanmingense*	0.036	*Segatella albensis*	0.004	*Fusobacterium nucleatum*	0.0003
*Cardiobacterium hominis*	0.025	*Rothia dentocariosa*	0.004	*Corynebacterium suicordis*	0.0007
*Corynebacterium tuberculostearicum*	0.044	*Oribacterium asaccharolyticum*	0.003	*Corynebacterium pilbarense*	0.0006
*Dialister invisus*	0.015	*Natronoflexus pectinivorans*	0.001	*Campylobacter gracilis*	0.0003
*Eikenella halliae*	0.026	*Neisseria bacilliformis*	0.003	*Schaalia odontolytica*	0.000
*Enterobacter hormaechei*	0.036	*Metamycoplasma salivarium*	0.001	*Mogibacterium. neglectum*	0.0007
*Fusobacterium periodonticum*	0.019	*Micrococcus antarcticus*	0.001	*Peptostreptococcus stomatis*	0.0003
*Corynebacterium matruchotii*	0.037	*Leptotrichia genomo * sp. *C1*	0.004	*Segatella maculosa*	0.0005
*Halothiobacillus neapolitanus*	0.018	*Klebsiella pneumoniae*	0.001		
*Leptotrichia shahii*	0.012	*Howardella ureilytica*	0.002		
*Atopobium rimae*	0.039	*Actinomyces massiliensis*	0.001		
*Leptotrichia wadei*	0.017	*Actinomyces odontolyticus*	0.005		
*Neisseria weaveri*	0.031	*Fusobacterium watanabei*	0.005		
*Parvimonas micra*	0.021	*Dialister pneumosintens*	0.001		
*Peptococcus niger*	0.018	*Curvibacter lanceolatus*	0.005		
*Prevotella micans*	0.034	*Conchiformibius kuhniae*	0.003		
*Pseudomonas lini*	0.039				
*Ruminiclostridium cellobioparum*	0.029				
*Segatella baroniae*	0.048				
*Selenomonas sputigena*	0.019				
*Treponema medium*	0.011				

**Table 2:** Results of the ALDEx2 (analysis of differential abundance taking into account sampling variation) test (**a**) at the genus level and (**b**) at the species level.

(**a**)		
Bacterial genus	we.eBH	Difference between P and C
*Kingella*	0.00069	7.56497
*Corynebacterium*	0.00820	4.77803
*Fusobacterium*	0.02174	−2.98425
*Haemophilus*	0.00717	5.50947
*Segatella*	0.01525	−5.16926
(**b**)		
Bacterial species	we.eBH	Difference between P and C
*Fusobacterium vincentii*	0.00751	−6.45941
*Kingella bonacorsii*	0.00104	7.42369
*Fusobacterium simiae*	0.00408	−7.07080
*Fusobacterium nucleatum*	0.02003	−3.53571
*Segatella maculosa*	0.00409	−5.21526
*Haemophilus parainfluenzae*	0.00772	5.74857
*Fusobacterium nucleatum*	0.00852	−7.15001

**Positive value** indicates **enhanced abundance** of bacterial genus and species (indicated in first table row) in **Permamem^®^ group,** while **negative value** indicates **enhanced abundance** in **Cytoplast™ group**. **we.eBH**—short for Wilcoxon Effect—Benjamini–Hochberg *p*-value; **P**—Permamem^®^ group; **C**—Cytoplast™ group.

## Data Availability

The original contributions presented in this study are included in the article. Further inquiries can be directed to the corresponding author.
